# The first case of multiple pulmonary granulomas with amyloid deposition in a dental technician; a rare manifestation as an occupational lung disease

**DOI:** 10.1186/s12890-018-0654-0

**Published:** 2018-05-22

**Authors:** Taizou Hirano, Tadahisa Numakura, Hiroshi Moriyama, Ryoko Saito, Yutaka Shishikura, Jun Shiihara, Hisatoshi Sugiura, Masakazu Ichinose

**Affiliations:** 1 Department of Respiratory Medicine, Tohoku University Graduate School of Medicine, 1-1 Seiryou-machi, Aoba-ku, Sendai, 980-8574 Japan; 2 Department of Respiratory Medicine, Hiraka General Hospital, Yokote, Japan; 3 Department of Respiratory Medicine, Nishi-Niigata Chuo National Hospital, Niigata, Japan; 4 Department of Anatomic Pathology, Tohoku University Graduate School of Medicine, Sendai, Japan; 5 Department of Respiratory Medicine, Saitama Medical Center Jichi Medical University, Saitama, Japan

**Keywords:** Dental technician, Amyloid deposition, Pulmonary granulomas, Silica

## Abstract

**Background:**

Occupational lung diseases, such as pneumoconiosis, are one of the health problems of dental workers that have been receiving increasing interest. Pulmonary amyloidosis is a heterogenous group of diseases, and can be classified into primary (idiopathic) and secondary (associated with various inflammatory diseases, hereditary, or neoplastic). To date, the development of pulmonary amyloidosis in dental workers has not been reported.

**Case presentation:**

A 58-year-old Japanese female presented with chest discomfort and low-grade fever that has persisted for 2 months. She was a dental technician but did not regularly wear a dust mask in the workplace. Chest X ray and computed tomography revealed multiple well-defined nodules in both lungs and fluorodeoxyglucose (FDG)-positron emission tomography revealed abnormal FDG uptake in the same lesions with a maximal standardized uptake value (SUV [max]) of 5.6. We next performed thoracoscopic partial resection of the lesions in the right upper and middle lobes. The histological examination of the specimens revealed granuloma formation with foreign body-type giant cells and amyloid deposition that was confirmed by Congo red staining and direct fast scarlet (DFS) staining that produce apple-green birefringence under crossed polarized light. Because there were no other causes underlying the pulmonary amyloidosis, we performed electron probe X-ray microanalysis (EPMA) of the specimens and the result showed silica deposition in the lesions. Based on these results, we finally diagnosed the patient with pulmonary granulomas with amyloid deposition caused by chronic silica exposure. Afterward, her symptoms were improved and the disease has not progressed for 2 years since proper measures against additional occupational exposure were implemented.

**Conclusions:**

Our case presented three important clinical insights: First, occupational exposure to silica in a dental workplace could be associated with the development of amyloid deposition in lung. Second, EPMA was useful to reveal the etiology of amyloid deposition in the lungs. Last, proper protection against silica is important to prevent further progression of the disease. In conclusion, our case suggested that occupational exposure to silica should be considered when amyloid deposition of unknown etiology is found in the lungs of working or retired adults.

## Background

Occupational and environmental lung diseases are a broad group of pulmonary disorders caused by the inhalation of harmful dusts, chemicals, or proteins [1] and are one of the health problems among dental workers receiving increasing interest [2, 3]. Working with dental abrasives and polishing materials can result in chronic exposure to various harmful materials such as silica, alloys, and acrylic plastics [2–5]. Pneumoconiosis is a well-known pulmonary disease in dental workers [2–5]. Although various types of pneumoconioses have been reported [2–6], to our knowledge, the development of pulmonary amyloidosis has not been reported. Here, we describe the first reported case of a dental worker who developed multiple granulomas with amyloid deposition in the lung, which may have resulted from long-term exposure to silica in the workplace.

## Case presentation

**Fig. 1:**
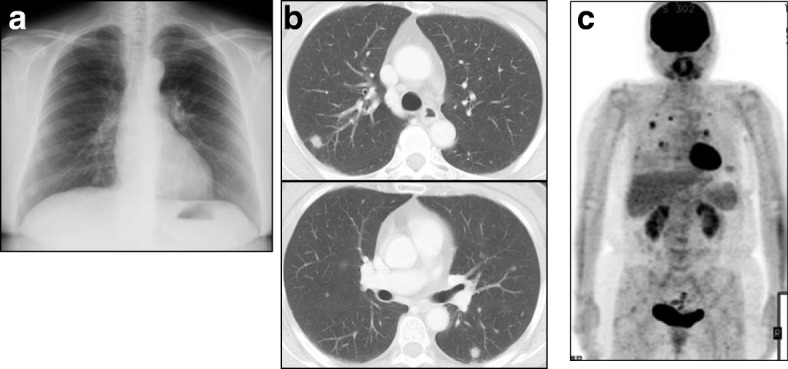
**a, b** Chest X ray (**a**) and chest computed tomography on admission (**b**) revealed multiple well-defined nodules in both lungs. **c** Fluorodeoxyglucose (FDG)-positron emission tomography revealed abnormal FDG uptake in the same lesions with a maximal standardized uptake value (SUV [max]) of 5.6

**Fig. 2:**
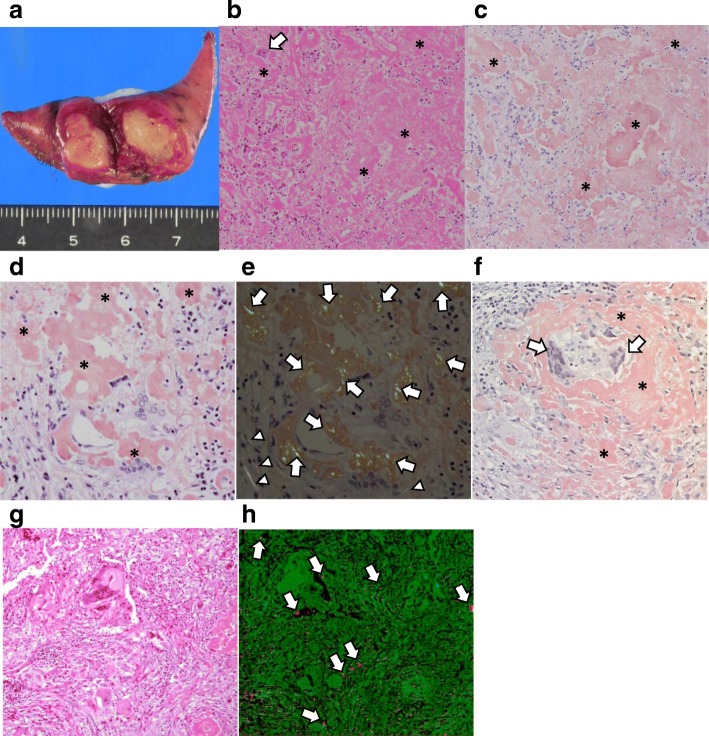
Histopathological findings of the lung nodules. **a** Macroscopic examination of a white hard nodule in the right middle lobe. **b** Low-magnification image of a hematoxylin and eosin (HE) stained specimen revealed granuloma formation with foreign body-type giant cells (arrow) and mild and focal chronic inflammatory changes with eosin-positive deposits (asterisk). **c** and **d** The images of direct fast scarlet (DFS) staining revealed that the deposits were DFS positive (asterisk) (**c**, at lower-magnification, and **d**, at higher-magnification). **e** The DFS staining-positive lesions produced an apple-green birefringence under crossed polarized light (arrows) (× 100). The collagen fibrils appeared white (arrow heads). **f** The deposits were Congo red positive (asterisk). Foreign body-type giant cells were detected (arrows) (× 100). **g** and **h** A representative photograph of HE stained specimen (**g**) and its image of electron probe X-ray microanalysis (EPMA) (**h**). A two-dimension EPMA-wavelength dispersive spectrometer (WDS) image of an elemental map corresponding to the area shown in (**h**), showing orange dots indicating silica (Si) accumulated in giant cells (arrows). The distribution of amino nitrogen was colored green (**h**)

A 58-year-old Japanese woman presented with chest discomfort and low-grade fever that had persisted for 2 months. She was a dental technician but did not regularly wear a dust mask in the workplace. She previously had a smoking history of 2 pack/year and no medical history. On admission, her body temperature was 37 °C. Physical examination results were almost normal. The laboratory examinations were also normal including blood smear differentials, C-reactive protein, serological tests for autoimmune disorders and malignant and hematological disorders, and serological tests for infectious diseases (Table 1). Chest X ray and computed tomography revealed multiple well-defined nodules in both lungs (Fig. 1a and b) and fluorodeoxyglucose (FDG)-positron emission tomography revealed abnormal FDG uptake in the same lesions with a maximal standardized uptake value (SUV [max]) of 5.6 (Fig. 1c). Then, we performed thoracoscopic partial resection of the lesions in the right upper and middle lobes (Fig. 2a). Histological examination of the specimens revealed foreign body-type giant cells and mild and focal chronic inflammatory changes with the infiltration of lymphocytes and plasma cells together with eosin-positive deposits (Fig. 2b). The deposits were direct fast scarlet (DFS) staining positive and produced an apple-green birefringence under crossed polarized light (Fig. 2d and e). Congo-red staining was also positive (Fig. 2f). Based on these results, we concluded that the substance in the lesion was compatible with amyloid. Although we diagnosed the patient with pulmonary amyloidosis, there was no obvious underlying causes that could typically cause pulmonary amyloidosis, including rheumatoid arthritis, primary Sjögren’s syndrome or multiple myeloma. We also excluded plasma cell neoplasm and multicentric Castleman’s disease because there was no infiltration of atypical plasma cells nor formation of multicentric lymphoid follicles in the lesions. Consequently, we focused on her occupational history and performed electron probe X-ray microanalysis (EPMA) of the specimens to evaluate the cause of pulmonary amyloidosis. EPMA of the specimens revealed the deposition of silica throughout the granulomatous lesions as well as among giant cells (Fig. 2g and h). From these results, we finally diagnosed the patient with multiple lung granulomas with amyloid deposition caused by chronic silica exposure. Afterward, her symptoms improved and the disease has not progressed for 2 years since proper measures against additional occupational exposure were implemented.

**Table 1:** Results of  laboratory examinations on admission

Blood count	
WBC	5800/μl
Neut	56.7%
Eos	3.6%
Bas	1.0%
Mo	4.9%
Ly	33.8%
RBC	430 × 10^4^/μl
Hb	13.0 g/dl
Plt	28.4 × 10^4^/μl
Arterial blood gas (room air)	
pH	7.427
PaO_2_	94.5 Torr
PaCO_2_	36.8 Torr
HCO_3_^-^	23.7 mEq/l
BE	−0.3 mEq/l
Blood chemistry	
TP	8.2 g/dl
Alb	5.0 g/dl
T-bil	0.3 mg/dl
AST	16 IU/l
ALT	16 IU/l
LDH	178 IU/l
ALP	242 IU/l
γGTP	16 IU/l
BUN	17.8 mg/dl
Cre	0.6 mg/dl
Na	143 mEq/l
K	4.0 mEq/l
Cl	108 mEq/l
Ca	9.9 mg/dl
Serum	
CRP	0.02 mg/dl
IgG	1952 mg/dl
IgA	309 mg/dl
IgM	95 mg/dl
IgG4	25 mg/dl
CEA	1.96 ng/ml
CA19-9	2.0 U/ml
ANA	x 40
Anti CCP antibody	< 0.6 U/ml
anti SS-Aantibody	negative
Anti SS-B antibody	negative
Serum amyloid A	2.9 μg/ml
s-IL-2R	412 IU/ml
β-D glucan	< 5.0 pg/ml
*Cryptococcus* antigen	negative
*Aspergillus* antigen	negative
*Candida* antigen	negative
T-SPOT	negative
Serum protein electrophoresis	
M protein	negative
Urine protein electrophoresis	
Bence-Jones protein	negative

*Neut* neutrophil, *UA* uric acid, *CRP* C-reactive protein, *CEA* carcinoembryonic antigen, *CA19-9* carbohydrate antigen 19-9, *CCP* cyclic citrullinated peptide, *s-IL-2R* soluble interluekin 2 receptor

## Discussion and conclusions

Occupational lung diseases are caused by exposure to various toxic materials in the workplace environment. The inhaled dust particles are deposited around terminal bronchiole to their neighborhood alveoli due to their size and air speed causing lung injury and inflammation by the released oxidants, enzymes, growth factors, and other inflammatory cytokines [1]. Dental workers are exposed to various types of dusts such as silica, aluminum oxide and heavy metals when polishing and grinding prosthetics and during casting operations. Such exposure can increase the risk of occupational lung disease [2–6]. Silica, which was identified in our case, is associated with a variety of occupational lung diseases including silicosis, tuberculosis, obstructive lung disease, and lung cancer. Thus, Japan Society for Occupational Health recommends that the maximum allowable concentration (MAC) of silica is less than 0.03 mg/m^3^ [7]. However, the concentration of silica in the workplace environment of dental workers sometimes exceeds the MAC. For example, one study showed that the concentration of silica in the air of the work environment of dental workers to be as high as 0.051 mg/m^3^ [6]. The most common occupational lung disease caused by silica exposure is pneumoconiosis [8, 9]. Most cases develop without symptoms, whereas cough, shortness of breath and fever may develop in severe cases [8, 9]. Although various types of pneumoconioses have been reported, the development of pulmonary amyloidosis in dental workers has not been reported according to our PubMed search.

Amyloidosis is a heterogeneous group of diseases characterized by the deposition of Congo red and DFS positive amyloid fibrils in the extracellular matrix of organs such as lung [10, 11]. Radiological findings of pulmonary amyloidosis can be typically classified into several clinical types, such as showing a diffuse alveolar septal pattern, single or multiple pulmonary nodules and tracheobronchial amyloidosis [10, 11]. The diagnosis of pulmonary amyloidosis sometimes requires surgical lung biopsy to confirm the histopathological findings with Congo red staining, which produces an apple-green birefringence under crossed polarized light. Pulmonary amyloidosis can be classified into primary (idiopathic) and secondary (associated with various inflammatory diseases, hereditary, or neoplastic) [9, 10]. In the present case, there was no obvious cause underlying the pulmonary amyloidosis. Therefore, taking into account the patient’s occupation and the histological findings of foreign body-type giant cell granulomas in the lung, we performed EPMA for further evaluation.

EPMA with a wavelength dispersive spectrometer (WDS) is a method of mineralogical analysis that can qualitatively and semi-quantitatively analyze the chemical composition of solid materials found in human tissue histology [12]. Takada et al. analyzed 162 cases of suspected occupational and environmental lung diseases by EPMA-WDS, and concluded that EMPA-WDS was useful to confirm the causes of various occupational and environmental lung diseases [12]. Although it is unknown whether the inhaled silica was a direct cause of the pulmonary amyloidosis, our histological as well as EPMA findings (Fig. 2) suggest that the chronic exposure to silica in a dental office could be associated with chronic inflammation, resulting in the development of amyloid deposition in the lung. Furthermore, a recent scientific report demonstrating that exposure to silica could increase the production of Congo-philic amyloid deposition in *C. elegans* also supported our findings [13]. Because there is no proven specific treatment for such patients, clarifying the occupational cause is key to prevention with effective occupational safety measures, such as good ventilation in the laboratory and personal protective devices, to avoid further progression of the disease.

In summary, we report the first case of a dental worker who developed multiple granulomas with amyloid deposition in the lungs likely caused by silica exposure in the workplace. Our case presented three important clinical insights: First, chronic exposure to silica in dental workplace could be associated with the development of amyloid deposition in lung. Second, EPMA was useful to reveal the unknown etiology of amyloid deposition in lung. Last, proper protection against silica is important to prevent further progression of the disease. In conclusion, occupational exposure to silica should be considered when amyloid deposition of unknown etiology is found in the lungs of working or retired adults.

## Abbreviations

EPMA: Electron probe X-ray microanalysis
FDG-PET: Fluorodeoxyglucose (FDG)-positron emission tomography (PET)
HE: Hematoxylin and eosin
MAC: Maximum allowable concentrations
SUV [max]: Maximal standardized uptake value
WDS: Wavelength dispersive spectrometer
